# It's all starting to come together

**DOI:** 10.7554/eLife.09853

**Published:** 2015-08-05

**Authors:** Lindsay A Becker, Aaron D Gitler

**Affiliations:** Department of Genetics, Stanford University School of Medicine, Stanford, United States and the Stanford Neurosciences Program, Stanford University School of Medicine, Stanford, United Stateslabecker@stanford.edu; Department of Genetics, Stanford University School of Medicine, Stanford, United Statesagitler@stanford.edu

**Keywords:** phase separation, protein aggregation, chaperones, prion-like protein, P body, stress granule, human, *S. cerevisiae*

## Abstract

Chemical, genetic and cell biology tools have been used to probe which RNA-protein granules behave like liquids and which behave like solids.

**Related research article** Kroschwald S, Maharana S, Mateju D, Malinovska L, Nüske E, Poser I, Richter D, Alberti S. 2015. Promiscuous interactions and protein disaggregases determine the material state of stress-inducible RNP granules. *eLife* **4**:e06807. doi: 10.7554/eLife.06807

**Image** Yeast cells form short-lived stress granules when exposed to excessive heat

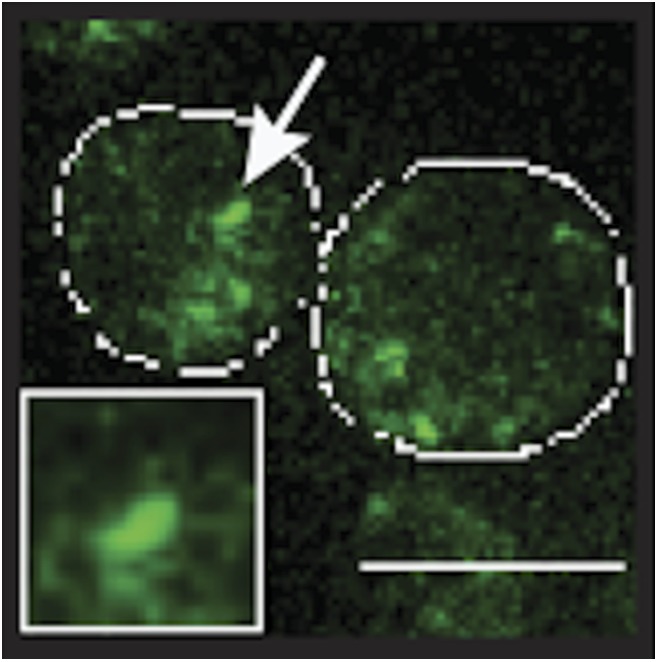

The cell is an extremely busy and crowded place. To cope with the huge variety of biological processes that occur at the same time and in very close quarters, cells divide their interiors into compartments. Some of these compartments – for example, the nucleus – are surrounded by a membrane. Others, such as the nucleolus, are not membrane-bound but still play critical roles that are essential for life.

Ribonucleoprotein (RNP) granules are non-membrane bound compartments that form from RNA molecules and RNA-binding proteins. Different classes of RNPs carry out diverse roles: for example, some can regulate gene expression while another (the nucleolus) produces ribosomes.

RNP granules self-assemble from soluble proteins and RNAs to form structures that constantly grow, shrink, and fuse. Recent studies suggest that some RNP granules behave like liquids (Brangwynne et al., 2009), and several researchers have hypothesized that RNP granules form by ‘demixing’ (kind of like how tiny droplets of vinegar separate from the olive oil and fuse to form larger droplets in a vinaigrette salad dressing). These and other findings have revolutionized how scientists think about the organization of compartments within the cell and about the biochemistry of protein accumulations (Li et al., 2012).

Stress granules and processing bodies (also called P bodies) are two kinds of RNP granules. Cells form stress granules in response to stresses such as excessive heat. Stressed cells halt the production of most proteins, and the molecular machinery needed to build these proteins (including the mRNA transcripts) coalesces into short-lived stress granules in the cell's cytoplasm (Kedersha and Anderson, 2002). When the stress is resolved, these stress granules dissolve, and protein production is resumed. But if the stress persists, the mRNA transcripts within the stress granules can be transferred to P bodies and broken down (Parker and Sheth, 2007).

In order for the chemical reactions that degrade RNA to occur within P bodies, the compartments must provide a highly flexible environment (where molecules can freely move around). Stress granules, on the other hand, act more like storage depots. This means stress granules are unlikely to need to be as flexible as P bodies. But stress granules must still be able to readily dissolve once the stress is resolved so that their constituent parts can be reactivated.

Now, in eLife, Simon Alberti from the Max Planck Institute of Molecular Cell Biology and Genetics and colleagues – including Sonja Kroschwald as first author – present a comprehensive analysis of the material states of stress granules and P bodies and uncover some unexpected differences (Kroschwald et al., 2015).

Kroschwald et al. used an alcohol called hexanediol to distinguish between RNP granules with liquid-like and solid-like states in living cells. Hexanediol interferes with weak hydrophobic (‘water-hating’) interactions between molecules. Since liquid-like states involve weaker interactions than solid-like states, it was reasoned that liquid-like states would be disrupted by hexanediol but solid-state ones would not. Indeed, when yeast cells containing fluorescently tagged P bodies and stress granules were treated with hexanediol, the P bodies rapidly dissolved. Furthermore, this was reversible; the P bodies rapidly returned after the hexanediol was washed out.

Kroschwald et al. then performed time-lapse microscopy and saw that the smooth, spherical P bodies often fused with one another and then relaxed back into a spherical shape. Other techniques further confirmed that molecules frequently entered and left P bodies (as would be expected in a weak, liquid-like state). These chemical and cell biological data suggest that the protein building blocks of P bodies interact weakly and move a lot, just like the molecules in a liquid.

The yeast stress granules were quite different and instead behaved like unstructured, solid aggregates. Unlike the P bodies, the yeast stress granules did not dissolve in hexanediol and had a rough surface. Furthermore, the misfolded protein aggregates that form when yeast cells are heat shocked overlap with the locations of stress granules, but not P bodies.

Kroschwald et al. hypothesized that stress granules could be a kind of useful aggregate and that misfolded proteins might help stress granules to form (Cherkasov et al., 2013). To test this hypothesis, they engineered yeast cells to produce protein fragments that can assemble into a large spherical particle within living cells. They then genetically fused various misfolded proteins to these fragments, resulting in particles that had misfolded proteins on their surface. After a heat shock, stress granule proteins were rapidly recruited to the particles. However, stress granules did not form on particles that did not have misfolded proteins on their surface. This finding suggests that misfolded protein aggregates can act like a scaffold, allowing stress granule proteins to bind and recruit other constituent parts to coalesce into a mature stress granule.

Unexpectedly, Kroschwald et al. observed that stress granules in mammalian cells appeared more liquid-like than the solid-like yeast stress granules (Figure 1). They propose that this is because yeast cells have robust aggregate-dissolving machinery that can readily dissolve stress granules from their solid state, whereas mammalian cells do not.

**Figure 1.**
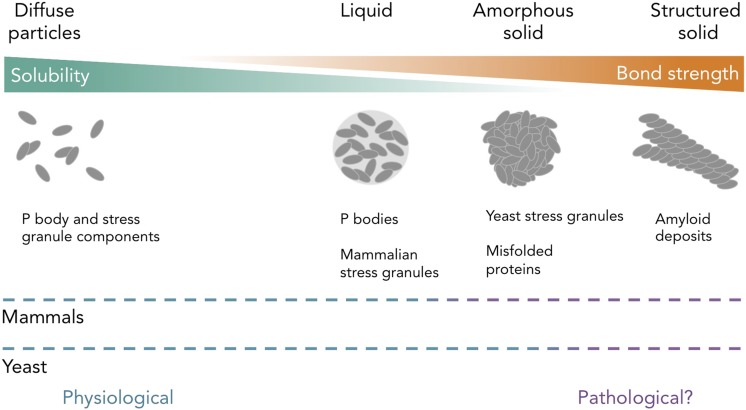
Protein and RNA accumulations can take on multiple forms within a cell. Kroschwald et al. report that, under healthy physiological conditions, P bodies in yeast and mammals and stress granules in mammals form liquid-like spheres (with weak bonds). Yeast stress granules form amorphous, unstructured solids, which are similar to aggregates of misfolded proteins. In several neurodegenerative diseases, insoluble deposits of proteins form structured assemblies with strong bonds, which are difficult to break down within cells. So-called ‘amyloid deposits’ are an example of this type of protein accumulation. These different states of matter are likely within a continuum, and the constituent components may undergo phase transitions to switch between different states under certain conditions. The color of the dashed lines indicates whether the states of matter on the continuum are considered healthy (“physiological”; blue) or potentially diseased (“pathological?”; purple) in mammals (top) and yeast (bottom). It is important to note that amyloid states in yeast (and potentially in mammals) can also be used for normal physiological processes.

These new results provide insight into a fundamental problem of cell biology, and may also be highly relevant to the development of several devastating human diseases. Neurodegenerative diseases, such as amyotrophic lateral sclerosis (ALS) and frontotemporal dementia (FTD), are associated with aggregates that contain some of the RNA-binding proteins that are also found in stress granules. Perhaps, in these neurodegenerative diseases, the nerve cells are killed when healthy RNP granules (with weak interactions) switch to a disease-causing insoluble state (with stronger interactions) (Li et al., 2013; Ramaswami et al., 2013). Indeed, Kroschwald et al. provide evidence that, in certain contexts, some proteins from P bodies and stress granules can form insoluble, immobile aggregates that are similar to those seen in these diseases.

Many questions lie ahead. How do P bodies and stress granules recruit their respective, distinct proteins given the weak and largely non-specific interactions that hold these components together? In mammalian cells, what stops liquid-like stress granules and P bodies from fusing? What controls whether different RNP granules take on liquid-like or disordered solid-like states? These and other questions are still unresolved and are an area of intense interest (Han et al., 2012; Kato et al., 2012). Characterizing the biochemical structures of protein accumulations, such as RNP granules, is not only relevant to our fundamental understanding of how cells work, it may also help unravel the causes of several neurodegenerative diseases.
